# Systematic review of applications and properties of the NoMAD instrument for assessing implementation outcomes: Study protocol

**DOI:** 10.3310/nihropenres.13559.1

**Published:** 2024-04-18

**Authors:** Tracy L Finch, Leah Bührmann, Sebastian Potthoff, Carl R May, Beckie Gibson, Jiri Gumancik, Oliver Wilson-Dickson, Melissa Girling, Tim Rapley

**Affiliations:** 1 Department of Nursing, Midwifery and Health, Northumbria University, Newcastle upon Tyne, England, NE7 7XA, UK; 2 Department of Social Work, Education and Community Wellbeing, Northumbria University, Newcastle upon Tyne, England, NE7 7XA, UK; 3 Faculty of Public Health and Policy, London School of Hygiene and Tropical Medicine, London, WC1H 9SH, UK; 4 Psychology, Northumbria University, Newcastle upon Tyne, England, NE1 8ST, UK

**Keywords:** NoMAD, Normalisation Process Theory measure, Normalisation Process Theory, NPT, Implementation Outcome measure, measure validation, systematic review

## Abstract

**Background:**

Implementation outcomes measures can be used to assess the implementation of complex health and social care interventions, but evidence for the use of these measures, and their psychometric properties, remains limited. The NoMAD ( Normalisation Me asure Development) survey, based on Normalisation Process Theory, was developed to assess, monitor, or measure factors likely to affect normalisation of a new practice from the perspective of participants who are engaged in an implementation process. Since publication in 2015, NoMAD has been translated into several languages and is increasingly being used in health and care research. This systematic review will identify, appraise, and synthesise the existing literature on the use of NoMAD as an implementation outcome measure, focusing on use and application across different studies and settings, and on its properties as a measurement tool.

**Methods:**

We will systematically search the bibliographic databases Web of Science, Scopus and PubMed for articles reporting empirical data in peer-reviewed journals. A citation search will also be undertaken in Google Scholar for primary NoMAD publications. Studies will be eligible for inclusion if they: (a) specify using NoMAD as a method and report results from using it, and/or (b) report a translation and/or validation study of NoMAD’s measurement properties. Screening of abstracts and full text articles will be done independently by two researchers. Data extraction will be structured to allow collection and descriptive synthesis of data on study characteristics, use of NoMAD, psychometric results, and authors’ reflections and recommendations.

**Conclusions:**

This review will provide the first synthesis of how NoMAD has been applied in health and care research, and evidence on its properties as an outcome measure since its publication. This will be used to update existing freely accessible guidance for researchers and other users, and disseminated through peer-reviewed publications, and engagement activities with researchers and practitioners.

## Plain language summary

**Background:** Implementation outcome measures are survey tools that have been developed to assess the success of implementation of health and social care interventions. Using theory, the NoMAD ( Normalisation Me asure Development) survey was developed to assess implementation processes, by asking structured questions of persons who are involved in a specific implementation. Once measures like NoMAD are used enough over time, and in a range of studies of different kinds of interventions in different settings, we can collate evidence from those studies to decide (1) how useful they are, and (2) how scientifically robust they are for making assessments in research. In this review, we will search the published literature for papers that report data from studies using NoMAD and summarise their characteristics and results to provide recommendations about how useful and scientifically robust NoMAD is at this time.

**Methods:**We will search databases (Web of Science, Scopus and PubMed), and a google search engine for published studies. We will include papers if they have used the NoMAD survey in their research and report results in their paper or have translated it into another language and tested it scientifically. Decisions about whether to include a paper will be made independently by two researchers, compared, and then agreed. A structured form will be used to capture the same information from each paper. We will summarise information on the studies, how they used NoMAD, what scientific evidence they provide about it, and what authors thought about using it.

**Conclusions:** This will be the first review of studies using the NoMAD survey since it was published in 2015. The results will be used to update publicly available guidance for researchers and other users. We will also share our findings directly through engagement activities with researchers and practitioners and will publish them in scientific journals.

## Introduction

Implementation science aims to advance our knowledge of how to optimally implement and sustain health and social care services and innovations. In this setting, understanding the impact and outcomes of implementation work relies in part on having useful and scientifically robust measurement tools that are appropriate to the kinds of outcomes that we aim to effect. Set out by Proctor *et al.* ^1^, implementation outcomes are distinct from service related outcomes and client outcomes, that have historically been included in clinical and applied health research designs that evaluate the impact of service interventions. They define implementation outcomes as *the effects of deliberate and purposive actions to implement new treatments, practices, and services* ^1^ (pg. 65). According to Proctor *et al.*, clinical or service outcomes cannot be achieved unless the intervention or treatment is implemented successfully.

The measurement of implementation outcomes is useful to the extent that reliable, valid, and robustly developed measures are available. Despite considerable advancement since Proctor’s framework in 2011 ^2^, the problems and challenges of advancing high quality approaches to measurement in implementation research for advancing theory development in the field ^3^ have been well documented. Systematically reviewing measures used for implementation outcomes in research on behavioural health care in 2015 ^4^ and updated in 2020 ^5^, Lewis and colleagues reported similar numbers of outcome measures deemed suitable for review (104, and 102 respectively). In the latter review, Mettert *et al.* ^5^ concluded that although there had been some improvement in measurement quality between reviews, few measures were identified as promising on the basis of the psychometric information provided. They noted that the rigorous development and testing of measures that are useful both for research and for practice, is still needed.

Whilst recognising the need for rigour, ‘pragmatic measures’ for implementation research ^6, 7^ have also been called for. These are measures that are rigorous but which also have utility for implementation science, in that they are important to stakeholders, have low burden for respondents and staff, are actionable, and are sensitive to change ^6^. A recent scoping review undertaken to define and conceptualise the concept of ‘pragmatism’ as a quality of implementation outcomes measures, indicated that there is currently still limited understanding of what pragmatism is and how it might be assessed for such measures ^8^.

One measure that has been developed for use in implementation outcomes research, and which aims to achieve a balance between theoretical underpinnings, psychometric rigour, and usability (or ‘pragmatism’) is the NoMAD survey instrument ^9– 11^. Developed to provide an adaptable measure of the constructs of Normalisation Process Theory (NPT) ^12^, NoMAD is designed to assess, monitor or measure factors likely to affect normalisation from the perspective of participants who are engaged in an implementation process. NPT is a middle range theory of implementation ^13^ that explains implementation processes as being revealed through the collective and collaborative activities of different groups of stakeholders involved in implementing service interventions in particular settings. This collective work centres around four distinct but related theoretical constructs: (1) ***coherence***, which is how individuals make sense of the intervention; (2) ***cognitive participation***, which is how individuals initiate and sustain their engagement in the process; (3) ***collective action***, as the process of working with the intervention to enact change; and (4) ***reflexive monitoring***, which refers to processes of appraisal of the effects of implementation activities and adaptation of processes as necessary. Developed through multiple methods of item generation, development and testing applied iteratively, and with different user groups ^10^, NoMAD provides a bank of 20 items that represent the four constructs of NPT. In an initial validation study ^8^, the theoretical structure of the four NPT constructs was confirmed in a sample of 413 NoMAD respondents using Confirmatory Factor Analysis (CFA). Internal consistency for the use of the 20 items to measure a general construct of normalization was α = 0.89 and ranged from α = 0.65 to 0.81 for the four related constructs. In its original form, NoMAD presents these items for rating on a five-point Likert scale from ‘strongly disagree’ to ‘strongly agree’. Given the highly context-dependent nature of implementation work, and of the variety of study designs and approaches that are applied to study it, the authors present NoMAD as a ‘pragmatic measure’ of implementation ^6^ and encourage creative and flexible use of it in relation to the users’ own implementation research and practice needs. At the time of publication, NoMAD was presented as demonstrating theoretical integrity and promise in terms of psychometric properties, but that further testing and reporting of the use of NoMAD in other studies and settings was required ^9^.

Since publication of NoMAD on the NPT website ( https://normalization-process-theory.northumbria.ac.uk/nomad-study/) in 2015, use of it in implementation research studies has steadily increased. There now exists a sufficient body of literature to undertake a systematic review of these studies to investigate the contribution that NoMAD is making to implementation outcomes assessment. NoMAD has now been included in several reviews of instruments for implementation research ^14– 16^, and is included as a measure in the online Implementation Outcomes Repository hosted by Kings Implementation Science. There are also a growing number of non-English translated versions of NoMAD that report on its psychometric properties ^17– 20^. This systematic review will therefore synthesise the existing literature on the use of NoMAD as an implementation outcome measure, focusing both on use and application across different studies and settings, and on its properties as a measurement tool.

### Aims and objectives

The overall aim of this systematic review is to identify, collate and synthesise the current evidence base from research that uses the NoMAD survey instrument in health, social care, and educational settings. The objectives of the review are to:

**Obj1:** Provide prospective users of the NoMAD tool with the first available summary of evidence of its properties as a research instrument, and evidence-informed guidance on how it can be used in research or in implementation work
**Obj2:** Advance theoretical understanding and further development of NoMAD as a measure of Normalisation Process Theory, through summarising empirical findings relating to NPT construct measures and associations of NoMAD with other measures as reported in the literature

We will address these objectives by investigating the following research questions:

**RQ1:** How has NoMAD been used in implementation research, what adaptations have been made within individual studies, and why?
**RQ2:** What NoMAD data is being reported, and what evidence is there for psychometric properties of NoMAD as a research instrument?
**RQ3:** What are users’ (authors’) reflections of NoMAD as a tool for understanding and measuring implementation processes?
**RQ4:** How does research using NoMAD inform the ongoing development of Normalisation Process Theory as a theory of implementation?

This review will be undertaken in parallel with a systematic review of qualitative NPT studies being undertaken by May *et al.* ^21^ to better understand how NPT explains the implementability, enacting and sustainment of complex healthcare interventions through its theoretical constructs. In May *et al.*’s review, research questions are included that will explore what NPT-informed qualitative studies identify in terms of mechanisms of implementation processes, and the different types of outcomes that are reported. The respective reviews include a subset of authors common to both, and the findings from each will inform the other in relation to the theoretical advancement of NPT.

## Methods

This systematic review will describe, appraise and narratively synthesise the findings of empirical research where NoMAD has been validated in a study, or used as part of the research design. The reporting of our methods is guided by the PRISMA-P guidance for systematic review protocols ^22^

### Patient and Public Involvement

Patient and Public Involvement was not considered appropriate for this study, as it is focused on synthesis of data from research papers about the use and scientific properties of a research instrument.

### Search strategy

Consistent with previous reviews similarly focused on specific tools or theories ^23, 24^, we will search three bibliographic databases (Web of Science, Scopus and PubMed) and a relevant search engine (Google Scholar) for articles reporting empirical data in peer-reviewed journals. A search string combining the concepts of “NoMAD / Normalisation” and “measurement instrument” will be used across databases (detailed below). Furthermore, articles citing any of the three key papers reporting the protocol ^11^, development ^10^ and validation ^9^ of the original UK-developed version of the NoMAD survey instrument will be identified using a citation search (Google Scholar), as well as the website reference that guided users of NPT and NoMAD, prior to publication of the study outputs ^25^. Known publications reporting the translation and validation of NoMAD to languages other than English ^18– 20, 26^, will also be included in the search strategy. This approach benefits from identifying contextually relevant source references that contribute to understanding users’ perspectives (RQ3 and RQ4) in relation to NoMAD, which would not be identified through keyword and mesh subject searching of article databases only. The database search will be checked against the citation search to ensure the quality of the bibliographic search string. This combined approach is expected to identify most published studies and reviews relevant for inclusion.

A large proportion of identified papers are likely to report study protocols. As indications of forthcoming research, these papers will not be included in the review, but will inform discussion of the review results. If considerable time passes between running searches and completing the review, we will also contact corresponding authors of study protocols to request any publications available for inclusion before finalising the review.

### Database search

The search string was developed using an adapted version of the PICO framework (Population, Intervention, Control, Outcome) for systematic reviews (see Table 1). The PICO concepts were operationalized in two search concepts (#1 “NoMAD/Normalization” and #2 “Survey”) that are to be combined in a search string (see Table 2).

**Table 1.** PICO Framework for NoMAD review.

**Population**	anyone using or administering the NoMAD survey (no restriction)
**Intervention**	NoMAD
**Control**	*not applicable*
**Outcome**	Normalization

**Table 2.** Search String for NoMAD review (PubMed).

#1 *NoMAD / * *Normalization*	"NoMAD"[Title/Abstract] OR ("Normalization Measure Development"[Title/Abstract]) OR ("Normalization Process Theory"[Title/Abstract]) OR ("Normalisation Process Theory"[Title/Abstract]) OR ("Normalization"[Title/Abstract]) OR ("Normalisation"[Title/Abstract]) OR ("Normalizing"[Title/ Abstract]) OR ("Normalising"[Title/Abstract])
#2 *Survey / Instrument*	"Survey"[Title/Abstract] OR "Instrument"[Title/Abstract] OR "Questionnaire"[Title/Abstract] OR "tool"[Title/Abstract] OR "measurement"[Title/Abstract] OR "measure"[Title/Abstract]
#1 AND #2	Total number for screening

### Screening

Screening on the full set of records identified will be conducted using Rayyan, software for management of systematic reviews. All papers will be assessed independently for inclusion by two members of the research team, who will discuss and resolve any discrepancies. A third reviewer will be referred to, if necessary, to achieve consensus for inclusion. This same process will be used for assessing full text articles.

### Inclusion

We will include:

- Any study that specifies using NoMAD as part of its methods (whether used in part or in full) AND reports empirical findings
- Any study that reports research to translate NoMAD into a non-English language

Where papers written in a non-English language are identified, we will seek to employ an appropriate bi-lingual reviewer to contribute to screening assessment and (if merited) data extraction from the paper, with English translation for the review.

### Data extraction

The data extraction form will be developed iteratively over a minimum of two rounds of piloting, involving multiple members of the research team, and undertaken using Excel software. A guide to data extraction, that provides additional information about the extraction questions and pre-set response options, will be developed to ensure consistency of data extraction amongst members of the authorship team. Approximately 10% of papers will be cross-checked by the lead researchers, for consistency and accuracy of data extraction, with further round(s) of cross-checking undertaken if significant inconsistences are identified.

***General use in research*.** Descriptive information will be extracted for all studies identified for inclusion. This will include authors, year of publication, country of lead author, paper type (intervention study, validation study), study design and methods, study sector and setting, the intervention implemented, what NoMAD version was used (language, number of items, response options), how and when (implementation phase) NoMAD was used, validation data if reported, any adaptations made to NoMAD, NoMAD scoring process and reported scores, authors’ reflections on benefits and limitations of NoMAD, and authors’ reflections on Normalisation Process Theory (NPT) for understanding implementation process and outcomes.

***Psychometric validation***. For a set of studies that report data relevant to psychometric assessment, including studies that report translation from English into another language, more detailed data extraction will be undertaken on individual properties. The Implementation Outcome Repository (IOR) hosted by Kings College London ^14^ contains 55 instruments for measuring outcomes in implementation research and includes information on and appraisal of NoMAD based on initial publications. To align with the approach of IOR and facilitate updating of its entry for NoMAD on completion of the review, we will use their approaches to psychometric and methodological quality appraisal for this set of studies to guide data extraction of these qualities. IOR uses the Contemporary Psychometrics Checklist (ConPsyCL) (developed by Kings psychometrics and measurement lab) to assess and provide information on the *psychometric quality* of measures included in the repository, on dimensions of reliability, validity and factor analysis. The IOR also uses the COSMIN checklist to assess three instrument quality domains: reliability, validity, and responsiveness ( *the ability of an outcome measure to detect change over time in the construct to be measured*). As we are not comparing multiple instruments, the concept of usability of the NoMAD tool will be assessed through authors’ own reports where available, in the publications.

A data extraction matrix will be developed for the full set of relevant psychometric quality and usability properties, to enquire and synthesise in relation to each study: (1) whether any evidence was collated/reported on the dimension; (2) authors’ own assessment on a dimension, if provided; and (3) the review team’s assessment against the guidance used in the IOR. The latter will be agreed between the review team members collectively, during an appraisal workshop to be undertaken once data is extracted.

### Synthesis of findings

The results section of the review will be organised around Research Questions 1-4.

In relation to *Use in implementation research (RQ1)*, we will present findings to describe the breadth of study designs and the different ways in which NoMAD has been used to date. Our initial publications ^9, 10^ advocated a ‘flexible’ approach to using NoMAD in different kinds of study designs, and explicitly encouraged adaptation and selective use of items *if appropriate*, depending on study-specific considerations such as the stage in an implementation process and roles of the survey participants in relation to the target intervention that is being implemented. In RQ1, we will explore how NoMAD has been used within mixed methods study designs, whether these have been cross-sectional or prospective, and how NoMAD has been conceptualised as a process or outcome measure in effectiveness study designs (Randomised Controlled Trials, Hybrid effectiveness-implementation trials). These findings will assist researchers to make evidence-informed decisions about study designs, and the potential contribution of NoMAD as a data collection tool. For example, where multiple time-points for data collection were included, how many were deemed sufficient, and what considerations were given to determining their frequency? In addition to research applications, the research team is keen to explore whether there have been other practical ways in which the NoMAD has been used – for example, with a focus on collecting insights to inform an ongoing implementation process such as for implementation or improvement work. Finally, we will describe the adaptations to the NoMAD survey itself (eg. numbers of items used, any changes to the response scale, or significant changes to the wording of items), that have been made by authors of the included studies.

Appraisal of emerging evidence on *psychometric properties of NoMAD as a research instrument (RQ2)* will enable a collated presentation of up-to-date evidence on quality indicators, across different language versions of NoMAD. This will help us to explore how transferable NoMAD appears to be in relation to the languages and practices of participants in different countries; and whether specific survey items are problematic, either on a meaning-based or a statistical level, in different countries or settings. This will assist users of NoMAD in different countries, and potentially avoid unnecessary duplication of translation work.

The review will provide the first opportunity to systematically investigate and collate *authors’ reflections of NoMAD* as a tool for understanding and measuring implementation processes (RQ3). We will synthesise authors’ reflections on their use of NoMAD, in relation to adaptations that have been made (described in RQ1). Furthermore, the information that can be learned from experiences of translating the tool into different languages is likely to be valuable for developing a more nuanced understanding of the items themselves, and how they may or may not provide a good representation of the theoretical constructs as provided in the original specifications of Normalisation Process Theory (NPT) ^12^.

Finally, we will synthesise and report the *conceptual and theoretical insights regarding NPT (RQ4)*, that are indicated by the empirical findings presented in the review. For example, the results of the quantitative studies included in the review may help us to explore questions about (statistical) relationships amongst the NPT constructs; relationships between NoMAD and other ‘implementation outcome’ measures ^2, 27, 28^ or in mixed methods studies, the triangulation of data collected using NoMAD alongside qualitative methods within single studies. We hope that authors will have engaged in theoretical reflection in relation to their study results, identifying ‘theoretically informative’ contributions of their work in using theory-based measurement approaches ^29^. We will also relate the findings of this review to more recent theoretical advancements of NPT where we have consolidated the constructs of NPT from across its various iterations over time ^30^, and from the qualitative synthesis of empirical findings of NPT studies that is currently underway ^21^.

### Quality assessment of included studies

As this review is primarily focused on synthesising (1) information about use of the NoMAD in research and (2) early findings from validation studies, we do not wish to make quality assessment of individual papers for the purpose of decisions about inclusion or exclusion in the review. Regarding the latter, quality appraisal will be undertaken during the synthesis stage of the review, with reference to COMSIN criteria, as used in Kings College’s IOR.

### Limitations of our approach

Focusing on ‘how NoMAD has been used in research’, will likely present a challenge in terms of heterogeneity of research designs, methods of use, interventions being implemented, and study contexts. This requires careful and detailed development work in planning data extraction with the synthesis in mind. An additional limitation is that the increasing pace of publication of studies that are relevant for inclusion in this review, means that the timing of our review will inevitably exclude forthcoming publications.

### Dissemination and impact

The findings of this review will directly inform future research using the NoMAD instrument, by providing an evidence synthesis that will aid researchers who are making decisions about what implementation outcomes to use in their study designs, and whether NoMAD meets their needs. Publication of the review may also prevent duplication of translation of NoMAD into other languages, where these translations already exist.

Consistent with our programme of work on NPT ^31^, our primary aim is to develop and make accessible through a number of formats, guidance on how to use NoMAD. The existing NPT website ( https://normalization-process-theory.northumbria.ac.uk/) has had high levels of user engagement since its establishment in 2015 ^31^, and users have been regularly accessing the original NoMAD survey and guidance on its use. This review will enable a thorough updating of the NoMAD guidance section of the NPT website, and provide an accessible access route for implementation practitioners, researchers and other users. To support dissemination to researchers looking for measures to assess implementation outcomes, we will request that our findings be reviewed for updating of the current entry for NoMAD in the Kings Improvement Science online Implementation Outcomes Repository (IOR) ( https://implementationoutcomerepository.org/). Finally, the review findings will directly inform our own ongoing programme of research and tool development on NoMAD and NPT, allowing future grant submissions to target clearly defined evidence gaps.

## Conclusions

As we continue to advance outcome measurement for implementation research, users of measurement tools require access to up to date evidence to inform their choices of tools, and application of them in their own research. Usability and appropriateness – pragmatics ^7, 16, 32, 33^ – of different measurement options, is as much a consideration as their psychometric properties. As a measurement tool informed by NPT ^9^, NoMAD has developed a sufficient body of literature that can be investigated and synthesised to further advance our understanding of NPT as a theory for understanding implementation processes and outcomes. This literature can inform the current and potential role of NoMAD as a measurement tool in implementation science. This systematic review will provide timely guidance to researchers and practitioners, on using NoMAD for implementation research and implementation practice.

## Data Availability

No data are associated with this article. Figshare: Northumbria University: PRISMA-P reporting checklist for ‘Systematic review of applications and properties of the NoMAD instrument for assessing implementation outcomes: Study Protocol’. Available at: https://doi.org/10.25398/rd.northumbria.25151810.v1 ^22^ Data are available under the terms of the Creative Commons Attribution 4.0 International license (CC-BY 4.0)
