# Supplementary material for: Systematic review of applications and properties of the NoMAD instrument for assessing implementation outcomes: Study protocol
Source: NIHR Open Res. 2024 Apr 18;4:21. [Version 1] doi: 10.3310/nihropenres.13559.1 (PMC11403288; doi:10.3310/nihropenres.13559.1)
Supplement: Peer review history

# Reviewer report: Reviewer response for version 1

**DOI:** 10.3310/nihropenres.14718.r32491

**Published:** 2024-09-17

**Authors:** Sze Ling Chan

**Affiliations:** 1 Health Services Research Centre, Singapore Health Services, Singapore, Singapore; 2 Health Services & Systems Research, Duke-NUS Medical School, Singapore, Singapore

NoMAD is a 20-item survey measuring the 4 constructs of the Normalization Process Theory. This is a protocol for a systematic review on the use of NoMAD since its publication in 2015. By summarizing how people have used it and their reflections on it, this review will provide valuable information on how to better support researchers to use NoMAD. This is a very well designed and written protocol with a careful consideration around each step of the systematic review process.

 Major comments: none

 Minor comments:

1. Some of the data collected (e.g. reasons for adaptations, authors’ reflections) are likely to be free text. The authors could elaborate on how they intend to present and summarize these results.

Is the study design appropriate for the research question?

Yes

Is the rationale for, and objectives of, the study clearly described?

Yes

Are sufficient details of the methods provided to allow replication by others?

Yes

Are the datasets clearly presented in a useable and accessible format?

Not applicable

Reviewer Expertise:

Implementation science, pharmacoepidemiology, health services research, pharmacogenomics

I confirm that I have read this submission and believe that I have an appropriate level of expertise to confirm that it is of an acceptable scientific standard.

# Reviewer report: Reviewer response for version 1

**DOI:** 10.3310/nihropenres.14718.r31735

**Published:** 2024-09-15

**Authors:** Fiona Riordan

**Affiliations:** 1 University College Cork, Cork, County Cork, Ireland

Thank you for the opportunity to review this interesting piece of work. With nearly 10 years elapsed since NoMAD was first made available, there is clear rationale for investigating how the tool has been applied and  adapted and to identify evidence of its psychometic properties, all of which will inform future development of the instrument. This review is being conducted in parallel with another review to investigate the application of NPT in qualitative studies, with scope for the reviews to inform one another and yield useful insights. The search strategy as set out in the protocol is robust, and the overall methodology is clear.

Is the study design appropriate for the research question?

Yes

Is the rationale for, and objectives of, the study clearly described?

Yes

Are sufficient details of the methods provided to allow replication by others?

Yes

Are the datasets clearly presented in a useable and accessible format?

Not applicable

Reviewer Expertise:

implementation science, mixed methods, health services research [diabetes management, integrated care]

I confirm that I have read this submission and believe that I have an appropriate level of expertise to confirm that it is of an acceptable scientific standard.

# Reviewer report: Reviewer response for version 1

**DOI:** 10.3310/nihropenres.14718.r32182

**Published:** 2024-06-27

**Authors:** Staci S Reynolds

**Affiliations:** 1 Duke University School of Nursing, Durham, USA

Thank you for the opportunity to review the manuscript “Systematic review of applications and properties of the NoMAD instrument for assessing implementation outcomes: Study protocol.” This is a well-written manuscript that provides a protocol for a future systematic review that will search for use of the NoMAD tool. Findings from this systematic review will assist researchers and clinicians by providing evidence on the use of and outcomes associated with the NoMAD tool, which can help strengthen future implementation science studies. Please see my specific comments below.

 Consider also searching the CINAHL database

 “This review will be undertaken in parallel with a systematic review of qualitative NPT studies being undertaken by May et al” – consider revising to not use “undertaken” twice in the same sentence

 Study Protocol

 Is the rationale for, and objectives of, the study clearly described? Yes, the overall purpose of this systematic review is to identify, collate and synthesise the current evidence base from research that uses the NoMAD survey instrument in health, social care, and educational settings. Specific objectives and research questions have been provided.

 Is the study design appropriate for the research question? Yes

 Are sufficient details of the methods provided to allow replication by others? Yes, sufficient details are provided regarding the methods used to allow for replication.

Is the study design appropriate for the research question?

Yes

Is the rationale for, and objectives of, the study clearly described?

Yes

Are sufficient details of the methods provided to allow replication by others?

Yes

Are the datasets clearly presented in a useable and accessible format?

Not applicable

Reviewer Expertise:

implementation science, infection prevention, quality improvement

I confirm that I have read this submission and believe that I have an appropriate level of expertise to confirm that it is of an acceptable scientific standard.
